# The Use of Dietary Supplements in Fitness Practitioners: A Cross-Sectional Observation Study

**DOI:** 10.3390/ijerph18095005

**Published:** 2021-05-09

**Authors:** Massimiliano Mazzilli, Filippo Macaluso, Stefano Zambelli, Pietro Picerno, Enzo Iuliano

**Affiliations:** Faculty of Psychology, eCampus University, 22060 Novedrate, Italy; massimiliano.mazzilli@uniecampus.it (M.M.); filippo.macaluso1@uniecampus.it (F.M.); stefano.zambelli@uniecampus.it (S.Z.); pietro.picerno@uniecampus.it (P.P.)

**Keywords:** dietary supplements, physical fitness, gyms, food supplements, body shaping, exercise

## Abstract

This study was aimed at evaluating the prevalence of use of dietary supplements (DSs) among gym users and gym instructors involved in body shaping-oriented fitness training. Furthermore, this study aimed to verify whether differences existed in the prevalence and in the types of DSs used in both gym users and gym instructors involved in body shaping-oriented fitness competitions vs. those not involved in fitness competitions. A survey was distributed to 316 participants, composed of 89 gym instructors and 227 gym users of both genders aged 27.3 ± 7.7. Among these participants, 52 were involved in competitions and 248 were not, while 16 participants did not specify either way. The results showed a high prevalence in the use of DSs in the population considered, with 85.4% of the participants declaring they used DSs, with high heterogeneity in the numbers and in the combinations used. No differences were found between gym instructors and gym users, or between participants involved and those not involved in competitions. The results indicate that DSs are widely used by persons involved in body shaping-oriented fitness training. The results also suggest that the majority of the participants decided individually which DSs to use.

## 1. Introduction

Dietary supplements (DSs) are products consumed in addition to a regular diet [1]. These products may include vitamins, minerals, amino acids, and a variety of other molecules. DSs are foodstuffs, but sometimes their commercialized pharmaceutical forms may be misleading for consumers, and consequently erroneously considered medicinal products. Additionally, the legislation regulating supplements is unclear, with discrepancies, for example, between regulations adopted in the USA, and regulations applied in the different countries within the European Union [2].

One thing is clear, though, however DSs are widely used in sport and physical training: depending on the type of sport and level of competition, the percentage of athletes that typically use DSs ranges from 40% to 100% [3]. Contrary to what one might believe, the use of DSs is not limited to athletes involved in competitions. They are also widely used by non-competitive sportsmen and women such as gym users, although without the necessity to succeed in competitions, the use of DSs might be thought to be less prevalent among gym users. This supposition, however, does not seem to be confirmed by scientific findings: the data contained in the literature suggest that the percentage of gym users using DSs is usually high. It is also possible to affirm that the overall prevalence of DS use by gym users is generally >40%. For example, a 44% prevalence was reported in a recent study carried out in Portugal [4], and a prevalence of up to 81% in a study performed in South Africa [5]. Differences and heterogeneity in the use of DSs among and within nations seem to suggest that local factors (e.g., national legislations, diets, socio-economic level, and exercise culture) could play an important role in determining the use of DSs in a gym context. Consequently, the results obtained in a specific geographic area cannot be generalized for use in a wider international context.

The literature also indicates various reasons for using DSs in a non-competitive context, including muscle gain, fitness/health amelioration, recovery time reduction, and the improvement of aesthetic appearance [4,5,6].

As regards the latter, it is important to highlight that fitness training aimed at body shaping consists of a series of methodologies conceived to improve muscular symmetry, while at the same time decreasing body fat percentage with the objective of “looking better”. This clarification is necessary because, typically, the studies outlined in the literature investigated the prevalence of DS use in bodybuilding athletes only, and not all persons involved in body shaping training consider themselves bodybuilders. There is a broader range of people who practice so-called “aesthetic workouts”; these workouts include but are not limited to bodybuilding workouts, and consequently, the data concerning the prevalence of DS use in bodybuilders cannot be directly related to persons performing fitness training, for whom no specific data on prevalence currently exists. 

Persons involved in bodybuilding have proven to be one of the groups with a very high prevalence of DS use with significant associated risks for health [1,7,8]. These potential health risks include severe cholestasis [9], liver damage [10] or kidney damage [11]. Despite the presence of several confounding factors, an increasing number of papers are focusing on the potential health risks that seem to be related to the presence of prohibited contaminants particularly found in bodybuilding supplements [12,13], and to the inappropriate use of DSs when not supervised by sports nutrition professionals [14].

The inappropriate use of DSs is probably associated with an underestimation of the risks due to inaccurate information [15,16]. In fact, athletes typically rely on trainers, or friends and family, for gathering information about the use of DSs [17]. Trainers/instructors must therefore be adequately informed to be able to provide accurate information on DSs, as underlined by Attlee et al. [18]. Unfortunately, instructors are not always properly informed about the proper use of DSs, and are sometimes DS users themselves. Consequently, investigating the prevalence and the pattern of DS use in fitness instructors may be of interest too. 

The aim of the present study was manifold: (**a**) to assess the prevalence of the use of DSs in persons practicing body shaping-oriented fitness; (**b**) to compare the prevalence and pattern of DS use between gym users and gym instructors; and (**c**) to compare the prevalence and pattern of DS use between persons involved in body shaping-oriented fitness competitions (included bodybuilding competitions) and persons practicing body shaping-oriented fitness training who are not involved in competitions. 

## 2. Materials and Methods

### 2.1. Study Design

The present study is a cross-sectional observation performed using a self-administered survey. The survey was conducted between December 2019 and February 2020.

### 2.2. Participants

Participants in the current study were enrolled through convenience sampling performed among persons from throughout Italy taking part in different national workshops for people passionate about fitness and body shaping training. For enrollment, the participants had to meet the following inclusion criteria: (1) to have been a gym user or instructor for at least 1 year; (2) to be involved in body shaping-oriented fitness training (including but not limited to bodybuilding); (3) to perform on average at least two hours of training/week (both for gym users and instructors) and to perform on average at least 8 h of work/week (instructors only); and (4) to have no medical conditions requiring the use of DSs as therapy. 

In total, 398 persons met the inclusion criteria; 82 persons refused to be involved in the study, and 316 persons declared their willingness to take part and signed an informed consent. The 316 participants who met the inclusion criteria and volunteered for this study were aged 27.3 ± 7.7 years old, with a range of 18 to 54 years old. There were 89 gym trainers (28.2% of the sample) and 227 gym users with no experience as instructors (71.8%). There were 52 participants involved in body shaping-oriented fitness competitions (16.5%) with 248 participants not involved in competitions (78.5%), and 16 participants (5.1%) who did not respond to the question. Of the participants, 83 were female (26.3%), and 233 were male (73.7%), with an average volume of training of 6.95 ± 3.08 h/week. In total, 192 participants were involved in weight training (60.8% of the sample), 111 in mixed training, including both weight and aerobic training (35.1% of the sample), and only 8 were involved in solely aerobic training (2.5% of the sample).

### 2.3. Procedures

An informal survey (i.e., not validated) was developed and administered to all participants in the study. The survey consisted of 14 items with multiple-choice answers investigating general information, experience of physical training and/or experience as a fitness instructor/personal trainer, involvement in body shaping-oriented fitness competitions, the type of training performed, the average weekly volume of training, and the use of DSs. 

All the items in the survey are reported in Table 1.

The survey responses concerning the use of DSs and the types of DSs used were utilized to assess the overall prevalence and pattern of DS use by all recruited participants. Responses concerning general information, experience of physical training and/or experience as a fitness instructor/personal trainer, involvement in sportive competitions, type of training performed, and average weekly volume of training were used to verify whether participants met the inclusion criteria, and to subsequently divide them into different categories (i.e., gym users or instructors, involved or not involved in competitions) so as to separately assess the prevalence for each category and thereafter perform statistical comparisons.

As regards item 11 (see Table 1), the following DSs were chosen as possible answers: vitamins, mineral salts, branched-chain amino acids, essential amino acids, creatine, whey proteins, and hydroxymethylbutyrate (HMB). These DSs were selected because they are the most commonly used in the fitness context [19]. In Table 2, a brief description is given of each DS under consideration.

### 2.4. Statistical Analysis

The data collected via the survey were firstly summarized using the descriptive analyses of count, percentage, mean, median and quartiles as the continuous variables, and of count and percentage as the categorical variables. The following variables were considered and summarized for the participants as a whole and for each separate category: (1) use of DSs (the prevalence of DS use), (2) types of DSs used, and (3) number of DSs used. The data were reported in an upset plot to allow a more detailed and easier interpretation of the data.

Then, different comparisons were performed to evaluate the presence of significant differences in these 3 variables among participants from the different categories: a non-parametric one-way Kruskal–Wallis *H* test (K-W *H* test) was used for continuous variables (number of DSs used), whereas a Chi-square test (*χ^2^* test) applied to the frequency values was used for categorical variables (the use of DSs and types of DSs used). For the analysis of continuous variables, a non-parametric analysis was used due to the non-normal distribution of the data previously tested. The comparisons were performed as follows: gym users vs. gym instructors (factor of *Gym Role*); persons involved in body shaping-oriented fitness competitions vs. persons not involved in any kind of competition (*Competition* factor). The data relating to these two comparative analyses were reported in upset plots to allow the better and easier interpretation of data.

For all the analyses, a *p*-value < 0.05 was considered significant. Bonferroni correction for multiple tests was used. All the analyses were performed using Microsoft Excel 365 for Windows, version 2012 (December 2020 (Microsoft Corp., Redmond, WA, USA)) and MedCalc for Windows, version 18.2 (MedCalc Software, Ostend, Belgium).

## 3. Results 

### 3.1. Results of Prevalence of Use of DSs and Results Relative to the Overall Sample

Concerning the use of DSs, 46 participants declared themselves to have not used DSs (14.6% of the sample), while 170 participants declared that they had (85.4% of the sample).

The three most widely used types of DS are listed here in order: whey proteins (187 participants declared using this DS, a figure equal to 59.2% of the sample), vitamin integrators (used by 137 participants, 43.4% of the sample) and branched-chain amino acids (BCAAs; used by 124 participants, 39.2% of the sample). Multiple answers were allowed for this item. 

In total, 20.3% of the participants declared using only one type of DS, 23.7% declared using two types of DSs, 16.1% declared using three types of DSs, and 32.7% declared using more than three types of DSs. 

**Figure 1:**
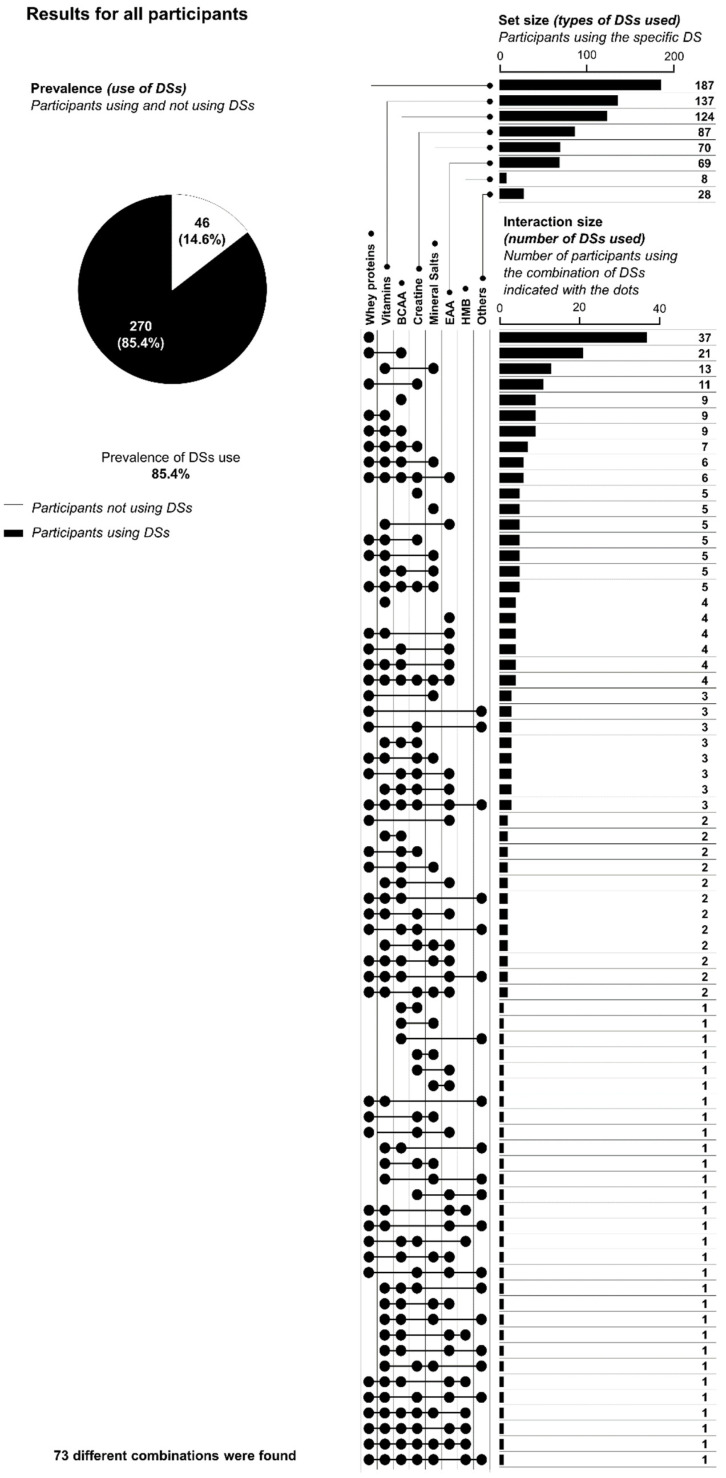
Use of dietary supplements (DSs) for all the participants. The pie chart reports the percentage of prevalence, whereas the upset chart reports the number of participants that declared use of a specific DS (upper part of the upset chart); the number of participants that declared use of a specific combination of DSs is indicated by the lined dots (lower part of the upset chart). BCAA = branched-chain amino acids; EAA = essential amino acids; HMB = hydroxymethylbutyrate.

The prevalence obtained from the survey is illustrated in Figure 1.

### 3.2. Results of Comparison between Gym Users vs. Gym Instructors

**Figure 2:**
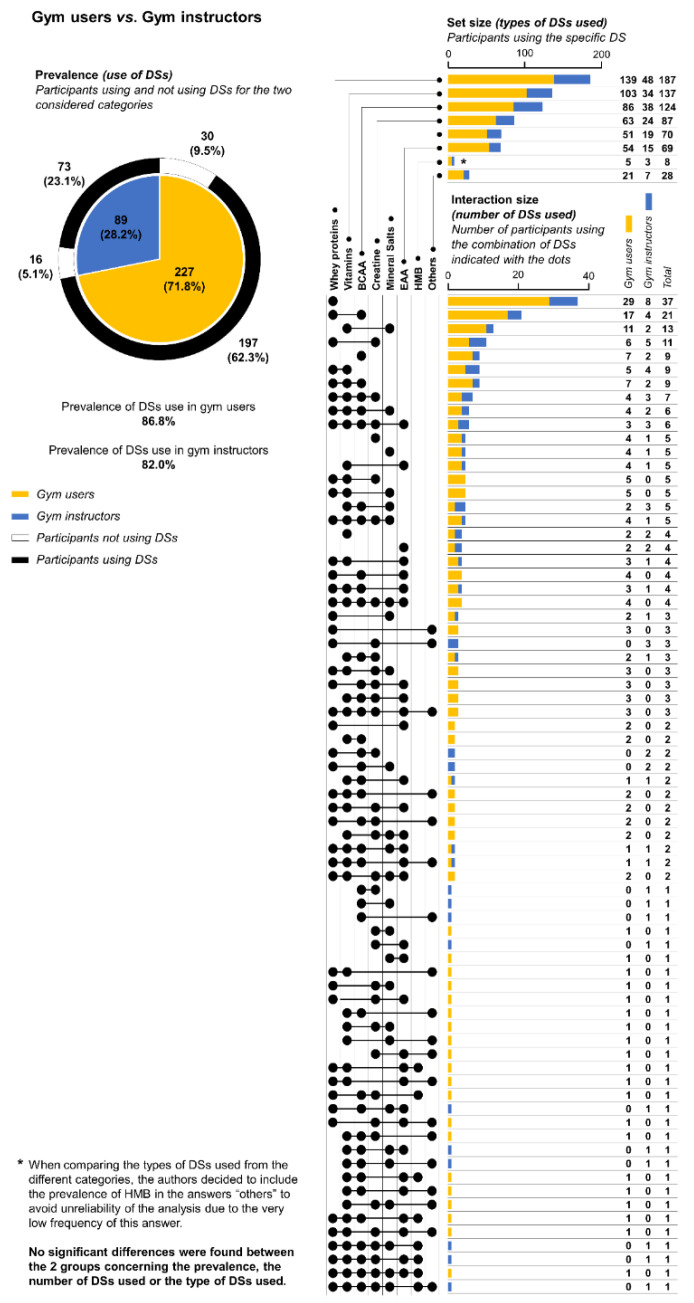
Results concerning the comparison of use of dietary supplements (DSs) between gym instructors and gym users. The pie chart reports the numbers of both gym users and gym instructors and their relative percentage compared to the total (inner part), and for each one of these two categories, the number of participants using or not using DSs was indicated (outer part). The upset chart reports the number of participants that declared the use of a specific DS (upper part of the upset chart), and the number of participants that declared the use of a specific combination of DSs is indicated by the lined dots (lower part of the upset chart), separated for gym users and gym instructors. BCAA = branched-chain amino acids; EAA = essential amino acids; HMB = hydroxymethylbutyrate.

No statistical differences between gym user and gym instructor were found in the three variables considered, i.e., the use of DS (*χ^2^_(1)_* = 2.273; *p* = 0.132), types of DSs used (*χ^2^_(6)_* = 2.261; *p* = 0.894), and number of DSs used (*H_(1)_* = 0.0002; *p* = 0.989). On average, each gym user used 2.68 DSs (first quartile = 2; median = 2; third quartile = 4), whereas each gym instructor was shown to use 2.67 DSs (first quartile = 2; median = 2; third quartile = 4). Detailed results and the upset plot are shown in Figure 2.

### 3.3. Results of Comparison between Participants Not Involved in Competition vs. Participants Involved in Competition

**Figure 3:**
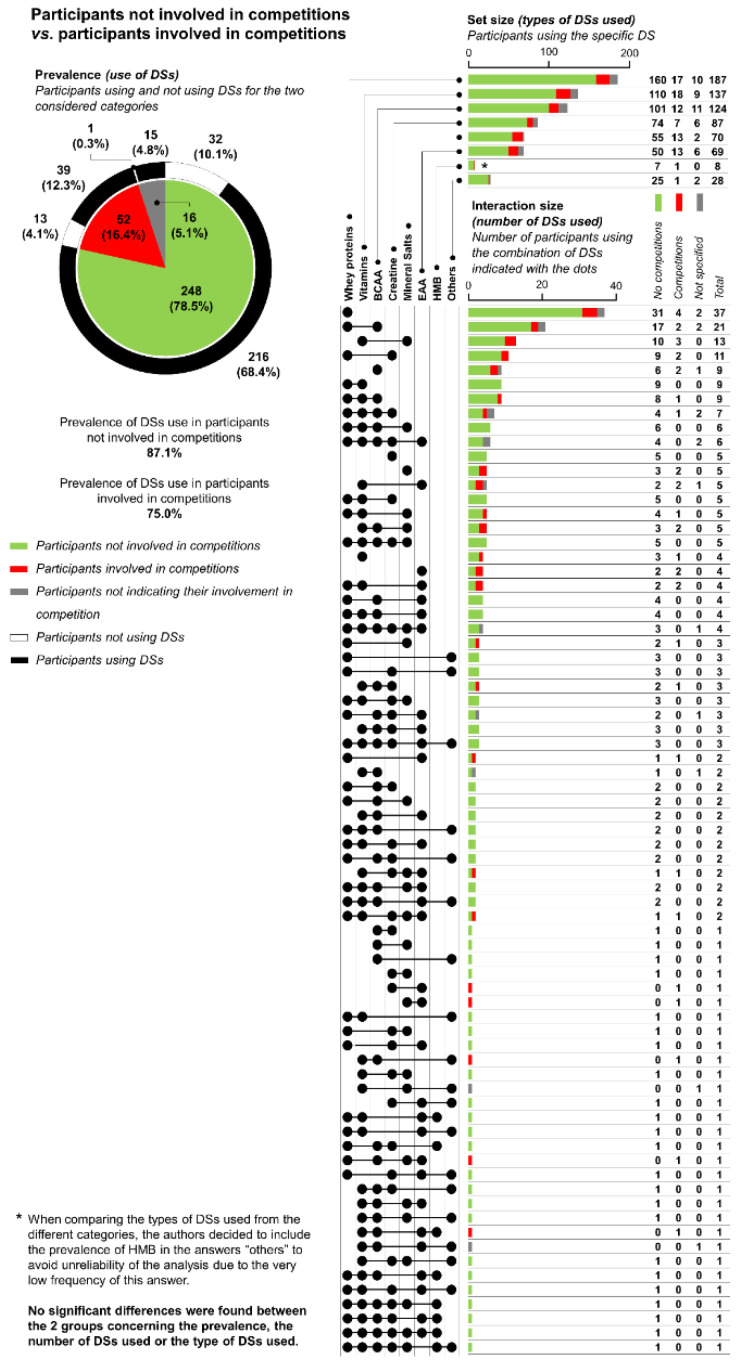
Results concerning the comparison in using dietary supplements (DSs) between participants involved in competitions vs. those not involved in competitions. The pie chart reports both the number of participants involved in competitions and the number of participants not involved in competitions, and their relative percentages compared to the total (inner part), and for each one of these two categories the number of participants using or not using DSs is indicated (outer part). The upset chart reports the number of participants that declared the use of a specific DS (upper part of the upset chart), and the number of participants that declared the use of a specific combination of DSs is indicated by the lined dots (lower part of the upset chart), divided for participants involved in competitions vs. participants not involved in competitions vs. participants that did not indicate their involvement in competitions. BCAA = branched-chain amino acids; EAA = essential amino acids; HMB = hydroxymethylbutyrate.

As shown in Figure 3, no statistical differences were found in the comparison between participants involved and those not involved in competition for the three variables considered, which were the use of DS (*χ^2^_(1)_* = 4.917; *p* = 0.027 that is not significant after Bonferroni correction), the types of DSs used (*χ*^2^_(6)_ = 11.107; *p* = 0.085), and the number of DSs used (*H_(1)_* = 3.474; *p* = 0.062). On average, each participant not involved in competition was shown to use 2.73 DSs (first quartile = 2; median = 2; third quartile = 4), whereas each participant involved in competition was shown to use 2.22 DSs (first quartile = 2; median = 2; third quartile = 4). Detailed results and upset plot are reported in Figure 3.

## 4. Discussion

As regards the main purpose of the present study, the results indicated a high prevalence of DS use among the populations considered. The participants that declared to not use DSs amounted to only 14.6% of the sample, whereas the participants who declared to use DSs made up 85.4% of the sample. This prevalence is high, especially when compared with the 44% prevalence recently obtained by Ruano and Teixeira [4] or the 49% prevalence obtained by Abu Mweis et al. [23], but it is similar to the results obtained by Senekal et al. [5] in South Africa (81% prevalence). However, it is necessary to consider that the participants of the present study are persons involved in body shaping-oriented fitness training, and therefore not representative of all gym users or gym instructors. This means that it was not possible to perform a direct comparison with other studies of literature that generally investigated the prevalence of DS use in overall gym users, in elite athletes, or in bodybuilding.

In any case, a comparison of the prevalence values alone is not sufficient to understand the differences among different populations in the use of DSs: in fact, the different types of DSs used, as well as the quantity and modalities of DS use, should be compared to gain a clearer perspective. Furthermore, as stated in the introduction, local factors such as national regulations, typical diets, socio-economic level, and exercise culture could play a significant role in determining the use of DSs in a gym context, and consequently, the differences between the results of the present study and the results obtained in other countries should also be considered whilst taking local factors into account.

By looking at the data reported in Figure 1, Figure 2 and Figure 3, it is nevertheless possible to observe a very high number of combinations of DSs used by the participants; in fact, in the present study, 73 different combinations of DSs were reported for 270 participants. There are a variety of explanations for these data. In the authors’ opinion, the most probable is that no guidelines are followed by participants in using DSs, and consequently the participants arbitrarily decided the types of DSs to use. It is in fact important to note that specific medical conditions or diseases requiring the consumption of DSs were considered as exclusion criteria for participation in the study, so it is improbable that this heterogeneity in the variety of combinations consumed can be due to the specific needs of the participants.

Another aspect to have emerged from the study was the significant increments in DS use registered over the last few decades in the geographical areas taken into consideration in the present study; in fact, a study performed 10 years ago by Bianco et al. [24] reported a 30.1% prevalence of protein supplementation use in a population of persons practicing strength training recruited in the same country as that of the present study. The prevalence reported by Bianco et al. [24] is much lower than the percentage obtained in the present study; protein supplementation was shown to be largely used by participants in the present study, and in particular, whey proteins (the most widely used DS in the present study) were shown to be used by 187 participants, indicating a prevalence of 59.2% (almost double the prevalence reported in Bianco’s study). Furthermore, 91 participants (28.8% of the total sample) declared using both whey proteins and BCAAs. This result seems to be consistent with the increase in DS use reported in other countries worldwide. This hypothesis is also corroborated by the constant growth of the DS market [25,26]. FederSalus, an Italian association of producers and distributors of DSs, indicated in a report from 2019 [27] that the food supplement market in Italy had reached a value of approximately EUR 3.6 billion (retail price value), having risen by 3.6% from 2018. These data seem to be confirmed by other national associations. The same report stated that Italy was the leading market for food supplements in Europe, and that this market represented 27% of the total European market. Other key information to take from this report includes that, in 2019, 32 million people in Italy used supplements (65% of the adult Italian population) for a variety of different reasons. With a view to analyzing local factors, this information seems to be particularly important as it suggests a generally high use of DSs in Italy (not only in gym practitioners). It is important to underline, however, that a direct comparison in the prevalence of DS use between the general population and the participants in the present study should be made with caution, as food supplements used by the general population include several products that are not generally used in a fitness context, and are therefore not included in the present study (e.g., herbs, oil supplements, fibers, probiotics, etc.). Furthermore, the general population will often use DSs for reasons different to those of gym users, such as for the prevention or treatment of health-related issues, the integration of nutrients, and compensation for deficiencies in specific substances.

The insignificant differences between gym users and gym instructors in all of the three variables considered seem to corroborate the hypothesis that a possible relationship between these two categories exists in the context of determining the use of DSs during training programs. Attlee et al. [18], in an investigation performed among gym users from a university community, stated that almost 60% of DS users used the internet as their main source of information on the use of dietary supplements, followed by family/friends at 42.1%. It was also reported in the same study that gym instructors were contacted by 40% of participants to obtain information on the use of DSs. The results of this study do not contradict these findings; as previously affirmed, the increased heterogeneity in the combination of DSs used seems to confirm that the use of DSs was subjectively decided by each participant, irrespective of being a gym user or an instructor. These data also allow for an attempt at answering a fundamental question: are gym instructors adequately informed about the correct and safe use of DSs? The most likely answer is no, but other investigations are necessary to properly answer this question, as well as for evaluating the real influence of instructors over gym users concerning the use of DSs. In any case, data reported in the study of Attlee et al. [18] indicate that medical physicians and dietitians represent a source of information on the use of DSs in only 15.7% and 12.8% of cases, respectively. Evidently, this is too low a percentage.

Concerning the role of competitions, no significant differences in DS use were found between participants involved and those not involved in competitions. This result was unexpected. It is particularly surprising that, despite the lack of significant differences after Bonferroni correction, the data seem to suggest that the prevalence of DS use by participants not involved in competition was higher than by participants involved in competitions. A possible explanation for this result may be that participants involved in competitions tend to have a lower prevalence of DS use, so as to avoid unintentional doping [28]; in fact, the presence of a banned substance in DSs can occur for different reasons (cross-contamination, poor manufacturing process, or even intentional contamination arising from unclear legislation), representing a significant risk for athletes undergoing anti-doping tests, considering that rates of DS contamination have been reported to range from 12% to 58% [29]. In this regard, a comparison with other Italian sporting populations might prove useful, though difficult to perform, as there is a high heterogeneity in DS use in athletes according to which sport and level of competition they are involved in. Moreover, the few recently available studies and data on the sporting population refer to very specific types of athletes (e.g., elite cyclists, amateur). It is, however, still interesting to note that an Italian Ministry of Health report from 2017 on doping controls reported that, of the total number of controlled athletes, 72.8% declared that they used DSs or other non-proscribed medicine [30]. This prevalence is lower than that obtained in the present study. In some way, these data also seem to confirm that athletes probably used DSs with more caution in order to avoid unintentional doping. A second possible explanation might be that athletes and persons involved in competitions have a higher level of knowledge of DSs and are consequently more able to avoid unnecessary DSs. The latter explanation, however, seems to be contradicted by a recent study investigating the DSs used by Italian beach-volleyball athletes. This study reported a high heterogeneity in supplementation habits in this sporting population, in accordance with the results of the present study [31]. This heterogeneity found in Italian beach-volleyball athletes would seems to suggest that other Italian sporting populations do not follow guidelines in using DSs either. In any case, beach-volleyball athletes cannot be representative of all the sporting populations, and consequently, a generalization of these result is not possible.

The large diffusion of these products necessitates the consideration of potential risks and/or benefits resulting from the use of these products. The first thing to clarify is that the use of DSs should not be considered positive or negative in an absolute sense: DSs are an important tool for improving the health of persons in need [32], and, as regards athletes or persons engaged in physical training programs, it has been demonstrated that DSs can also play a positive role in improving energy, optimizing exercise training adaptations and/or promoting recovery in certain instances [19]. Nevertheless, it is important to understand that the incorrect use of DSs can endanger health for reasons such as possible overuse, interactions between different DSs taken together, interactions of DSs with other medical substances, the contraindication of some DSs, and/or contaminations of DSs [13,33,34]. Several studies have reported an unfortunately high propensity to underestimate the potentially adverse consequences of these products [35,36], and this may be why people choose to manage the use of DSs by themselves, acquiring information from unreliable sources such as those previously stated [4,37]. This approach is highly dangerous because a great number of athletes and gym users do not have enough knowledge and information to manage the use of these products safely and effectively. This is why, as recommended by many health institutions such as the Food and Drug Administration (FDA; https://www.fda.gov/consumers/consumer-updates/fda-101-dietary-supplements, accessed on 5 May 2021) and the National Institutes of Health (NIH; https://ods.od.nih.gov/factsheets/WYNTK-Consumer/, accessed on 5 May 2021), it is best to consult a healthcare professional before taking DSs. With exercise, a proper use of DSs can be highly useful in cases where there is a specific need (e.g., deficiency of a specific vitamin or mineral) or when it is necessary to rationally support the physiological and biological adaptations induced by particular training programs (e.g., sustaining muscle hypertrophy processes or promoting post-exercise recovery). Incorrect use can, however, produce health risks both in the short and long term. Another risky “habit” is to arbitrarily consume specific DSs to achieve hypothetical benefits obtained by other persons; people are not all the same, and what is of benefit to one person may not be of benefit to another, and may even be harmful, due to biological or physiological differences.

Other interesting data that emerged from the present study concerned the main types of DSs used by the participants. The second most frequently used type of DS was shown to be the vitamin supplements used by 137 participants, equivalent to 43.3% of the sample. There is no evidence concerning the efficacy of vitamins used for improving sports performance or muscle building; most vitamin supplements did not lead to an improvement of performance in athletes without a vitamin deficiency, and sometimes the use of some vitamins reduced athletic performance, negatively influencing the intracellular adaptations and body composition [19,38]. BCAA supplements were the third most frequently used DS (used by 124 participants, 39.2% of the sample), despite limited evidence existing to support their efficacy in supporting resistance training—the role of BCAA in increasing muscle protein synthesis seems to be related principally to the role that leucine plays [39], but despite the results of some studies seeming to suggest that BCAAs can promote recovery, alleviate soreness, attenuate the reduction in force production and reduce the rate of perceived exertion in prolonged exercise [19,40], the literature lacks strong evidence to support BCAA supplementation during resistance training, especially for the promotion of muscle protein synthesis as well as the enhancement of sporting performance [19,41]. Whey protein supplements were shown to be the most used DS (used by 124 participants, 39.2% of the sample), and their use seems to be more supported by scientific literature: a systematic review with meta-analysis performed in 2018 [42] reported that protein supplementation (not only whey protein but also soy, casein, milk, or mixed protein supplementations) significantly enhanced changes in muscle strength and size during prolonged resistance training in healthy adults. The review also indicated that the efficacy of protein supplementation is generally improved by training experience, whereas aging reduces the efficacy of this type of DS. In any case, protein intake (including protein supplementations) greater than ~1.6 g/kg/day does not further contribute to an inducement of gains in fat-free mass in persons involved in resistance training programs. The efficacy of protein supplementation in improving muscle building has also been confirmed in another review [19], but its authors also indicate that the overuse of protein supplements can be dangerous to some people’s health [43]. At any rate, the literature does not seem to indicate that whey proteins are more effective than other types of protein supplements.

Finally, one last comment should be made on the use of HMB (hydroxymethylbutyrate). Kerksick et al. [19] indicated that, currently, the efficacy of only four types of DS is supported by strong scientific evidence, and these DSs are HMB, creatine monohydrate, essential amino acids, and protein supplements. In previous paragraphs, it was reported that protein supplements (whey proteins) were shown to be the most widely used DS by participants involved in the present study, while creatine was used by 87 participants (27.5% of the sample), and essential amino acids were used by 69 participants (21.8%); HMB, despite the presence of evidence concerning its efficacy, was shown to be very rarely used by participants of the present study (8 participants, 2.5% of the sample). For this reason, the authors decided to include the prevalence of HMB in the answer “Others” to avoid the unreliability of the analysis related to the low frequency of this answer.

## 5. Conclusions

The present study seems to indicate an overall high prevalence of DSs in gym users and instructors involved in body shaping-oriented fitness training, with no differences between participants involved and those not involved in competitions. The source of information of participants was not investigated in the present study, but the high prevalence of DS use suggests that further investigations into this area are necessary. A healthcare professional, as recommended by many health institutions, should be consulted before beginning any course of DS to reduce the health risks associated with incorrect use of these products. It is important to not underestimate the risk of DS self-administration, but to rely on expert evaluation, as is has normally done in other aspects of fitness assessment [44,45].

## Figures and Tables

**Table 1:** Items of the survey.

	Items	Possible Answers
1	Please, indicate your gender.	Male Female
2	Please, indicate your age.	*Indicate the number*
3	Do you have any medical condition requiring the use of dietary supplements as therapy?	Yes No
4	Are you a gym user involved in body shaping-oriented workouts?	Yes No
5	If so, for how long?	Less than 1 year More than 1 year
6	How many hours do you train a week?	*Indicate the number*
7	What kind of training do you practice?	Resistance/weight training Aerobic/cardiovascular training Mixed training Other
8	Do you usually take part in body shaping-oriented fitness competitions?	Yes No
9	Do you use dietary supplements for training?	Yes No
10	How many dietary supplements do you use?	*Indicate the number*
11	Please, indicate the type of dietary supplements you use. *(multiple choices are allowed)*	Vitamins Mineral salts Branched-chain amino acids Essential amino acids Creatine Whey proteins Hydroxymethylbutyrate (HMB) Others
12	Do you work as a gym instructor involved in body shaping workouts?	Yes No
13	If so, for how long?	Less than 1 year More than 1 year
14	If you are a gym instructor, how many hours a week do you work as an instructor?	*Indicate the number*

**Table 2:** Description of the dietary supplements chosen as possible answers to item 11 of the survey.

Dietary Supplements	Brief Description
Vitamins	Vitamins are essential organic compounds necessary to regulate several metabolic and neurological processes, and normal cell function. Consequently, in the field of physical exercise, the potential benefits attributed to the supplementation of these compounds are various [19]. Vitamins are available both as multivitamin and single-vitamin preparations.
Mineral salts	Minerals are essential inorganic elements. They have structural roles in tissue, and they are important components of enzymes and hormones, as well as being regulators of metabolic and neural control. Consequently, in the field of physical exercise, the potential benefits attributed to the supplementation of these elements are various [19]. Minerals salts are available both as multimineral and single-mineral preparations, and sometimes supplements contain both vitamins and minerals (multivitamin/mineral supplements).
Branched-chain amino acids (BCAAs)	BCAAs consist of three essential amino acids (leucine, isoleucine, and valine). The metabolism of BCAAs is involved in some specific biochemical muscle processes. The potential benefits attributed to BCAA supplementation during training include the improvement of muscle protein synthesis, the alteration of glucose metabolism, the attenuation of muscle damage, and the reduction in perceived fatigue [19,20].
Essential amino acids (EAAs)	EAAs consist of nine amino acids (histidine, isoleucine, leucine, lysine, methionine, phenylalanine, threonine, tryptophan, and valine). As EAAs include BCAAs, the potential benefits of EAAs during exercise are similar to those of BCAA. In fact, some authors have suggested that the effects of EAA ingestion are due precisely to the BCAA content [21]. On the contrary, other authors have more recently affirmed that EAA supplements can significanlty stimulate muscle protein synthesis compared with BCAA alone [19].
Creatine	Creatine is an organic compound, and its role is to store energy (creatine phosphate). In the field of exercise training, creatine supplementation is used to increase high-intensity exercise capacity and muscle mass [19].
Whey proteins	Overall, protein supplements are consumed to obtain EAAs to support muscle growth, maintenance, and repair during training programs. In particular, whey proteins (or serum proteins) are proteins isolated from the whey of cow’s milk, and they consist of α-lactalbumin, β-lactoglobulin, proteose peptone, serum albumin, and immunoglobulins. Whey protein supplements are commonly used during exercise programs because the digestion and the absorption of these proteins are rapid compared with other proteins [22].
Hydroxymethylbutyrate (HMB)	HMB is a metabolite of the leucine. In the field of physical exercise, HMB supplements are used to increase muscle mass and to activate muscle protein synthesis [19].

## Data Availability

The data presented in this study are available on request from the corresponding author.
